# Construction and validation of the ultra-short version of the Parenting Scale (PS-4)

**DOI:** 10.3389/fpsyg.2026.1595258

**Published:** 2026-02-27

**Authors:** Bjarne Schmalbach, Dirk Baier, Yvonne Krieg, Elmar Brähler, Sören Kliem

**Affiliations:** 1 Medical Psychology and Medical Sociology, University Medical Center of the Johannes Gutenberg University, Mainz, Germany; 2 Institute of Delinquency and Crime Prevention, Zurich University of Applied Sciences, Zurich, Switzerland; 3 Faculty of Law, University of Zurich, Zürich, Switzerland; 4 Criminological Research Institute of Lower Saxony, Hannover, Germany; 5 Clinic and Polyclinic for Psychosomatic Medicine and Psychotherapy, Rostock University Medical Center, Rostock, Germany; 6 Department of Psychosomatic Medicine and Psychotherapy, University Medical Center of the Johannes Gutenberg University Mainz, Mainz, Germany; 7 Ernst-Abbe-Hochschule Jena – University of Applied Sciences, Jena, Germany

**Keywords:** assessment, child development, mental health, parenting behavior, scale development

## Abstract

Parenting behavior is a central determinant of childhood development and is thus deserving of more scientific attention. In the present article, we constructed an ultra-short scale for the assessment of parenting styles, the Parenting Scale 4 (PS-4). To this end, we analyzed large samples of parent–child dyads—one representative of the German general population (Sample 1), the other representative for the German federal state Lower Saxony (Sample 2). We applied an algorithm-based scale-shortening technique in Sample 1 and confirmed the resulting model in Sample 2, finding excellent model fit and—given the extreme brevity—acceptable reliability. Furthermore, we show that the model is invariant across parent and child genders. Correlations with the Strengths and Difficulties Questionnaire remain virtually unchanged compared to a longer version of the Parenting Scale, which is evidence for the PS-4’s validity. Overall, the PS-4 can be recommended for the assessment of parenting behavior, particularly in large-scale surveys with time constraints.

## Introduction

Parenting is one of the most important determinants of childhood development (Belsky, 1984; Sulik et al., 2015; Tran et al., 2017). A myriad of crucial outcomes is shaped by the way parents treat and teach their children: health, life satisfaction, and success later in life among many others (Abubakar et al., 2015; Hamilton, 2016; McLeod and Shanahan, 1993; Milevsky et al., 2007; Raboteg-Saric and Sakic, 2014; Steinberg et al., 1989; Waters, 2015).

The Parenting Scale (PS; Arnold et al., 1993) is one of the most widely applied parenting behavior assessment tools - also in the evaluation of trainings and prevention programs (de Graaf et al., 2008; Salari et al., 2012). The original PS consists of 30 items and can be divided into two factors: Overreactivity and Laxness. A third factor (Verbosity) was discussed in the beginning but has been refuted by subsequent research (for a review see Salari et al., 2012). Various shortforms of the PS have been developed over the years (Lorber et al., 2014; Reitman et al., 2001; Rhoades and O’Leary, 2007). The shortest that we are aware of is the 8-item version by Kliem et al. (2019). While eight items is very brief, even shorter scales—such as the Patient Health Questionnaire 4 (Kroenke et al., 2009; Löwe et al., 2010)—have been developed and enjoy great popularity. They are particularly useful in survey research, where every item counts and minimizing time investment is of the utmost importance.

The present study thus served the purpose of shortening the PS-8 to the ultra-short screening version PS-4. To this end, we first examined the psychometric properties of the PS-8 items and constructed a subset of 2 items per scale. We then tested this subset in a second, independent sample using confirmatory factor analysis (CFA). In addition, we tested for measurement invariance between genders and tested correlations with the Strengths and Difficulties Questionnaire (SDQ; Goodman, 2001). Since the PS is supposed to capture problematic parenting tendencies, we expect positive correlations between the PS-4 subscales and the difficulties subscales of the SDQ and positive correlations with the strengths subscale.

## Method

### Procedure and participants

Sample 1 was collected as part of a representative survey of the German general population in 2013. The demographic consulting company (USUMA, Berlin, Germany) which was responsible for data collection, initially contacted a total of 4,360 individuals based on random selection. The selection procedure uses three steps: 258 geographic regions in Germany, households within a specific region, and individuals in a specific household. Of those, many refused to provide any information (*n* = 591), were not present upon visitation (*n* = 563), refused to be interviewed (*n* = 540), or were not included for other pre-defined reasons (*n* = 158). A trained interviewer visited all participants. After making sure that they possessed sufficient command of the German language, the interviewer informed them of the general purpose of the study and obtained informed consent. In the case of minors, consent of the parents was also obtained. Following a sociodemographic interview, the participants completed self-report questionnaires in the presence of (but without any interference from) the interviewer. The final study sample consisted of *n* = 2,508 individuals. Out of those, 539 reported currently raising a child. Eight of these observations were excluded because of missing values. Thus, our exploratory analysis sample consisted of 531 observations. No systematic differences between participants with complete and incomplete data were observed. Given the low and largely unsystematic proportion of missingness, no imputation procedures were applied. The study was authorized by the Ethics Committee of the Medical Faculty of the University of Leipzig (Az.: 050/13-03.05.2013).

Sample 2, was collected as part of a representative survey of fourth graders in the German federal state of Lower Saxony, carried out by the Criminological Research Institute of Lower Saxony (Kriminologisches Forschungsinstitut Niedersachsen – KFN) as part of an evaluation study of the “Klasse2000” universal prevention program (Kliem et al., 2020a,b) and was funded by the Federal Centre for Health Education (Bundeszentrale für gesundheitliche Aufklärung – BZgA). The survey involved a random sample of year four classrooms in Lower Saxony from all classrooms taught in the 2016/2017 school year. In total, K = 740 classes were selected for the survey. The final sample was reduced to *k* = 528 classrooms based on refusals to participate from school principals or teachers; this corresponds to a classroom participation rate of 71.4%. Of the *N* = 9,880 students attending the participating classes, *n* = 6,376 students participated in the survey, which corresponds to a student participation rate of 64.5%. All 6,376 participating children received a questionnaire for their parents by “satchel post.” From 3,324 of these parents a completed questionnaire was sent back to the KFN. This corresponds to a response rate of 52.1%. In addition, we removed observations with missing values. Thus, our final sample consists of *n* = 3,133 observations. Only children whose parents returned a signed consent form to the teacher were allowed to take part in the interview. The ethics committee of the Georg Elias Müller Institute for Psychology at the University of Göttingen (N0. 184/2018) has given a positive ethics statement for this procedure. Furthermore, the survey was approved by the regional school authorities (Landesschulbehörde Niedersachsen H1Rb-81402-09-2017). During the entire survey, the children were guided through the eleven-page questionnaire by the test leaders using a manual. Participation is entirely voluntary among principals, teachers, parents, and children, and nonparticipation does not lead to any negative consequences. Children whose parents have not provided written informed consent are not surveyed. Teachers administered the written informed consent forms, did not forward them to the researchers, and destroyed them 2 weeks after the survey was conducted. Since the study design provides for complete anonymization, no signature of the participating adults could be obtained for their own participation. The sociodemographic variables of both samples are displayed in Table 1.

**Table 1:** Sociodemographic variables of both samples.

	Sample 1 (*n* = 531)		Sample 2 (*n* = 3,133)	
	*n*	%	*n*	%
Parent gender				
Male	220	41.4	345	11.1
Female	311	58.6	2,776	88.9
Parent age				
*M*, *SD*	39.56	8.30	42.08	5.54
Child gender				
Male	278	52.7	1,531	49.0
Female	250	47.3	1,592	51.0
Child age				
*M*, *SD*	8.92	5.23	2.69	0.58

### Instruments

The Parenting Scale 8 (PS-8, Kliem et al., 2019) consists of two subscales - overreactivity and laxness - measured with four items each. Respondents describe their typical reactions to certain behaviors a child can exhibit. All items are bi-polar, giving two potential extremes of the reaction spectrum. Respondents rate on a scale of 1 to 7 to what extent these responses are typical of them. Reliability coefficients for the present study are reported in Table 2.

**Table 2:** Correlation matrix of the Parenting Scales 4 and 8 as well as the strengths and difficulties questionnaire (Sample 2).

	1	2	3	4	5	6	7	8	9
1. Overreactivity 4	0.622								
2. Laxness 4	0.329	0.546							
3. Overreactivity 8	0.847/0.790^+^	0.271	0.645						
4. Laxness 8	0.387	0.895/0.854^+^	0.326	0.714					
5. Emotional Symptoms (SDQ)	0.165	0.093	0.164	0.109	0.696				
6. Conduct Problems (SDQ)	0.283	0.155	0.312	0.176	0.295	0.554			
7. Hyperactivity (SDQ)	0.209	0.112	0.244	0.137	0.329	0.498	0.791		
8. Peer Problems (SDQ)	0.147	0.107	0.114	0.138	0.383	0.307	0.291	0.676	
9. Prosocial Behavior (SDQ)	−0.203	−0.104	−0.233	−0.138	−0.098	−0.400	−0.277	−0.176	0.660

Internal consistency is displayed on the diagonal, ^+^adjusted for overlap in accordance with Levy (1967).

Sample 2 also included the Strengths and Difficulties Questionnaire (SDQ; Goodman, 2001; Klasen et al., 2003). This questionnaire consists of 25 items that assess a parent’s view of their child’s particular strengths and weaknesses in terms of behavior, self-regulation, and social behavior. There are five subscales: Conduct Problems, Emotional Symptoms, Hyperactivity, Peer Problems, and Prosocial Behavior. Response options range from 1 (*Not true*) to 3 (*Certainly true*). Reliability coefficients are reported in Table 2.

### Statistical analyses

We conducted all analyses in R, using the *EFAutilities*, *ezCutoffs*, *lavaan*, *matrixStats*, *moments*, *multilevel*, *paran*, *semTools*, and *stuart* packages (Bengtsson, 2020; Bliese, 2016; Dinno, 2018; Jorgensen et al., 2020; Komsta and Novomestky, 2015; Rosseel, 2012; Schmalbach et al., 2019; Schultze, 2020; Zhang et al., 2020). We excluded those respondents from the analyses who had not answered the PS-8 scale or had missing data on one or more of the items. We applied exploratory factor analysis and parallel analysis (PA; Horn, 1965) to Sample 1. The purpose of these procedures was to check how many components should reasonably be extracted from the eight indicators, and how the items load on them. We consider loadings of 0.50 and greater as sufficient. In addition, there should be no substantial cross-loadings (0.20). After this initial check we used *stuart*’s *bruteforce* algorithm to find the best 2 × 2 solution among all possible item subsets. “Best solution” here means best model fit and best composite reliability.

We then fitted the resulting model in Sample 2 using robust diagonally weighted squares estimation Li (2016) and delta parameterization. In, addition to the χ2 -test, we utilized the Comparative Fit Index (*CFI*), the Tucker-Lewis Index (*TLI*), the Root Mean Squared Error of Approximation (*RMSEA*), and the Standardized Root Mean Squared Residual (*SRMR*) to evaluate model fit. We judged these indices based on the traditionally recommended cutoff values: the *p*-value of the χ ^2^-test should be greater than 0.05 (0.01), *CFI*/*TLI* greater than 0.97 (0.95), RMSEA smaller than 0.05 (0.08), and SRMR smaller than 0.05 (0.10), respectively, to indicate good (acceptable) fit between theoretical model and empirical data (Schermelleh-Engel et al., 2003). To supplement these traditional fixed cutoff values, we employed the simulation-based approach introduced by Schmalbach et al. (2019). Here, the empirically derived fit values are compared to the fit index distributions obtained by simulating a large number of data sets with the same underlying parameter table and overall same properties as the empirical one. As generally recommended, we estimate reliability using McDonald’s ω (Dunn et al., 2014; Green and Yang, 2009, Formula 21). To test for measurement invariance between genders, we followed the guidelines provided by Wu and Estabrook (2016) and evaluated the comparisons using the recommended procedure (Chen, 2007). That is, we successively constrained item thresholds, factor loadings, item intercepts, and item residual variances to be equal between groups. For each of these steps Δχ ^2^ should be non-significant, Δ *CFI* should be smaller than 0.01 and Δ *RMSEA* should be smaller than 0.015.

## Results

### Item descriptive statistics and exploratory analyses (Sample 1)

We report item descriptive statistics in Table 3. All items were approximately normally distributed upon inspection of skewness and kurtosis (Kim, 2013). Parallel analysis showed clear evidence for two substantial components in the PS-8. The first three empirical eigenvalues were 2.62, 1.57, and 0.72 - compared to 1.24, 1.16, and 1.10 in the randomly generated data sets. Loadings from the subsequent exploratory factor analysis are also reported in Table 3. It should be noted that Items 5 and 8 had comparatively low loadings. In addition, Item 8 evinced a cross-loading. Next, we entered all items into the *stuart* to find the optimal two-factor solution among the possible 36 subsets. The best solution, which had excellent model fit, χ ^2^(1) = 0.667, *p* = 0.414, *CFI* = 1, *TLI* = 1.006, *RMSEA* = 0, *SRMR* = 0.006, is marked in Table 3.

**Table 3:** Item descriptive statistics of the Parenting Scale 8 (Sample 1).

	*M*	*SD*	Skewness	Kurtosis	λ F1	λ F2
Item PS1^O^	5.30	1.48	−0.66	2.58	−0.036	0.688
Item PS2^O, 4^	5.79	1.18	−1.05	3.90	0.092	0.737
Item PS3^L, 4^	5.19	1.45	−0.67	2.98	0.763	0.055
Item PS4^L^	5.13	1.51	−0.78	3.06	0.653	−0.043
Item PS5^R, O^	4.33	1.75	−0.16	2.00	−0.107	0.484
Item PS6^L, 4^	5.66	1.24	−1.08	4.18	0.769	0.030
Item PS7^R, O, 4^	5.82	1.43	−1.34	4.31	0.064	0.536
Item PS8^R, L^	5.57	1.35	−0.94	3.58	0.466	0.240

M, Mean; SD, Standard Deviation; λ, Standardized factor loadings on factors 1 and 2 in the exploratory factor analysis; R, Reverse-coded item; O, Overreactivity scale; L, Laxness scale; 4, Part of the PS-4.

### Confirmatory analyses (Sample 2)

We then tested the model generated in the EFA sample in the independent CFA sample. As can be seen in Table 4, fit measures indicated very good fit for the two-factor model and the χ ^2^-test was not significant at *p* = 0.134. Even the simulated cutoffs, which are in this instance much stricter than the traditional cutoffs, were not violated. The two latent variables correlated strongly, *r* = 0.581, *p* < 0.001. Reliability was $\omega_{\text{Overreactivity}}$ = 0.622 [0.601, 0.643] and $\omega_{\text{Laxness}}$ = = 0.546 [0.522, 0.568], respectively. In general, these coefficients would be considered small, even unacceptable. However, it should be noted that ultra-short scales tend to yield smaller reliability coefficients. Based on the Spearman-Brown prophecy formula, we can estimate that two four-item scales of the same general characteristics would evince ω s of 0.767 and 0.706, respectively. In order to confirm the two-dimensionality of the PS-4 scale, we then also tested the uni-dimensional version of the model. However, this model evinced unacceptable fit by all accounts.

**Table 4:** Model fit statistics of the Parenting Scale 4 (Sample 2).

	chi^2^	CFI	TLI	RMSEA	SRMR
2FM empirical fit	2.247	1.000	0.997	0.020	0.006
2FM simulated cutoffs	3.984	0.998	0.990	0.031	0.009
1FM empirical fit	242.516	0.914	0.741	0.196	0.060
1FM simulated cutoffs	5.830	0.998	0.994	0.025	0.011

Instances in which the empirical fit measure is worse than the simulated cutoff should be considered evidence of model misfit.

Next, we tested for measurement invariance between genders. This means that we considered both the child’s and the parent’s gender as well as the match/mismatch between the two. The results for these analyses are summarized in Table 1. As is evident from the non-significant increases in χ ^2^ - in addition to the virtually unchanged values for *CFI* and *RMSEA* - from step to step, the model can be considered to be invariant across groups of child gender, parent gender, and child–parent gender match/mismatch. Merely the *RMSEA* for the configural model of parent gender is substantially higher than in the more constrained models. This can probably be explained by the low number of free parameters, which is known to inflate *RMSEA* (Kenny et al., 2015). Overall, however, we find strong evidence for gender invariance. This is an important requisite for comparisons of both latent and observed means and variances between these populations (Gregorich, 2006).

### Correlations with the SDQ

Overall, we observed the expected pattern of correlations between the PS-4/−8 and the SDQ scales (see Table 2). Specifically, there were small to moderate positive correlations between the two subscales of the PS-4/−8 and the four problem-oriented scales of the SDQ. In contrast, the SDQ prosocial behavior scale correlated negatively with both PS subscales. In order to gauge the difference between the 8-item PS and the ultra-short PS-4, we first examined the correlations between the two versions of the scales. As is evident with the very high correlations, the majority of the PS-8 s variance is still explained by the PS-4. In investigating the differences between correlations of the long and short versions of the PS and the SDQ subscales this similarity becomes even clearer: For Overreactivity, the average difference was Δ *z* > −0.001, *p* = 0.999, while for Laxness the average difference was Δ *z* = −0.012, *p* = 0.992. This means, one can use half the items and apply the PS-4 instead of the PS-8 with a negligible loss of information and validity.

## Discussion

The aim of the research at hand was the construction of an ultra-short version of the PS. This was achieved by drawing on two large samples - one representative of the German general population, the other representative for the German federal state of Lower Saxony.

While a loss of reliability is close to inevitable while eliminating items, we observed exactly the decrease that was to be expected for a reduction from four to two items per scale. Model fit, on the other hand, improved greatly. All criteria evinced excellent goodness-of-fit. Furthermore, we found evidence for measurement invariance between genders: This means that the observed means can be compared both between different gendered children and parents. In addition, we examined whether the matching of same /different gendered children and parents could result in a lack of invariance, which was not the case. As is evident with the correlation matrix of the PS-4, PS-8, and SDQ, information loss was minimal in eliminating four items of the PS-8. Specifically, we observe the same pattern of correlations and correlational magnitudes for both the 4- and 8-item version: Positive correlations for the Prosocial Behavior scale and negative correlations for the other four scales, which deal with problematic tendencies.

### Limitations

The present study is limited in that we have not applied the final four PS-4 items in isolation. It appears unlikely that the outcomes of the psychometric investigation should change, but future studies should strive to replicate our findings to be sure.

As in all survey studies, the data may have been falsified by the respondents due to processes of social desirability or deliberate deception, whereby such processes can be avoided by informing the participants in detail about the underlying data protection mechanisms (e.g., anonymous survey as well as in sample 2 no feedback to parents, school or teachers) should be kept to a minimum. Response rates were = 57.5%/57.6%, which is quite common in general population studies (e.g., Kliem et al., 2014, 2015, 2018, 2021, 2024, 2025; Schmalbach et al., 2019) as well as in school surveys (Beckmann et al., 2021; Kliem et al., 2020a,b; Rehbein et al., 2015). However, it cannot be excluded that participants/students with a high level of stress often did not participate in the surveys. A further limitation concerns potential partner and co-parent influences in families with separated parents. Although the study focuses on the primary caregiver, child outcomes are likely shaped by complex interactions with non-resident parents and new partners (e.g., co-parenting quality, conflict, parenting practices in the other household). These influences could not be reliably measured or disentangled in the present design and may have introduced unobserved heterogeneity. Future studies should incorporate multi-informant and multi-household assessments to better capture interparental dynamics and their impact on child outcomes. Furthermore, Germany, as well as most other countries in northern Europe, has abolished any form of parental violence (physical as well as emotional, i.e., corporal punishment) as an accepted form of parental educational method. As of the year 2000 corporal punishment is considered abusive and hence a criminal offence under German law (§ 1,631 Abs. 2 *Bürgerliches Gesetzbuch* [German Civil Code]) which is not the case in several US states. It can therefore be assumed that German parents are more prone to giving socially desirable answers than their US counterparts. Parenting behaviors and disciplinary practices are embedded within cultural, legal, and normative contexts. The PS-4 was developed and validated using samples from Western, high-income countries, where parenting norms and legal regulations surrounding child-rearing are relatively similar. Its generalizability to cultural contexts with different normative parenting practices, legal frameworks, or social expectations cannot therefore be assumed. Future research should explicitly test cross-cultural measurement invariance and cultural validity before applying the PS-4 in non-Western or legally divergent contexts.

Lastly, the ultra-short format of the PS-4 entails a trade-off between measurement efficiency and content coverage. While the scale captures core dimensions of dysfunctional parenting, it cannot fully reflect the behavioral breadth and nuance assessed by longer versions of the Parenting Scale. Accordingly, the PS-4 is not intended to replace longer instruments such as the PS-8, nor is it suitable for individual-level assessment or clinical decision-making. Rather, it should be understood as an ultra-short screening instrument designed for contexts in which assessment time and respondent burden must be minimized, such as large-scale surveys and population-based research. Although the PS-4 demonstrates good model fit and evidence of construct validity given its minimal item count, longer versions remain preferable when more detailed assessment is required. Due to its limited reliability, associations involving the PS-4 may be attenuated and should be interpreted with appropriate caution.

## Conclusion

In sum, the PS-4 can be recommended as an ultra-short, yet valid and (given its extreme brevity) reliable assessment tool of parenting behavior. It is particularly useful in large-scale surveys where time is of the essence.

## Data Availability

The raw data supporting the conclusions of this article will be made available by the authors, without undue reservation.
